# Leveraging Community Health Worker’s Deep Community Knowledge to Address Food and Nutrition Security in Appalachia

**DOI:** 10.13023/jah.0704.05

**Published:** 2025-12-01

**Authors:** Alexis Kimbro Scott, Melissa D. Olfert, Dawn Brewer, Makenzie Barr, Julie Plasencia

**Affiliations:** University of Kentucky; West Virginia University; University of Kentucky; University of Kentucky; University of Kentucky

**Keywords:** Appalachia, Appalachian Region, community health workers, community health, food insecurity, rural health

## Abstract

**Introduction:**

Community health workers (CHWs) are trusted professionals in communities because of the deep understanding and cultural insights learned from their lived experiences. In Appalachia, CHWs are embedded in the community, giving them a unique understanding of how social determinants, like food and nutrition security, impact health outcomes.

**Purpose:**

This study aimed to examine the professional characteristics and knowledge of CHWs while identifying culturally sensitive practices around food insecurity.

**Methods:**

This cross-sectional, mixed-methods study used an online survey and in-depth phone interviews with CHWs in the Appalachian Region to examine their professional characteristics and knowledge on food security in their community. Quantitative surveys included a Cultural Competence Assessment (CCA) with two subscales: Cultural Awareness and Sensitivity (CAS) and Cultural Competence Behaviors (CCB). Surveys were analyzed descriptively to examine professional characteristics, while interviews underwent thematic analysis to identify community-based food and nutrition solutions.

**Results:**

Fourteen CHWs completed the online survey, and over 75% reported being able to provide guidance to families on food assistance programs. Findings from in-depth interviews highlight the challenges that families face in accessing these programs, including generational differences that affect access to home-cooked v packaged foods, as well as access to entitlement programs due to non-custodial caretaker status.

**Implications:**

The deep cultural competence among CHWs makes their partnerships a valuable community asset. Their unique position and knowledge of the community enable them to identify opportunities for community-based solutions to improve food and nutrition security in rural Appalachia.

## INTRODUCTION

Community health workers (CHWs) are uniquely poised to intervene on food and nutrition security issues in areas negatively impacted by social determinants of health in regions such as rural Appalachia. They are effective in their roles and can work alongside healthcare providers to educate and improve health outcomes,^1^,^2^ partly due to their historical knowledge and the earned trust of their community.^3^

Poverty and economic distress in rural Appalachia significantly impact access to affordable and nutritious foods, educational attainment, employment opportunities, and healthcare services.^4^,^5^ In Central Appalachia, which includes regions in Kentucky (KY) and West Virginia (WV), food insecurity rates are 17.5%, compared to the national average of 10.5%.^5^,^6^ This region also experiences two to three times higher mortality rates from chronic diseases compared to the national and non-Appalachian KY rates.^5^

Their position and expertise in the community present an opportunity to engage CHWs in addressing complex community challenges.^3^ However, the characteristics, skills, and knowledge of CHWs that contribute to their effectiveness in enhancing access to food and food assistance programs within community-based settings are not well studied. Therefore, this study examines CHWs’ professional characteristics and knowledge in identifying culturally sensitive practices related to food insecurity in rural Appalachia.

## METHODS

### Study Design and Participants

This study used a cross-sectional mixed methods approach of online surveys and in-depth phone interviews. The study was approved by the University of Kentucky Institutional Review Board #43445, and participants consented prior to commencement.

Recruitment and data collection occurred from January – May 2019, through two CHW organizations serving KY and WV in Appalachia. Eligibility criteria included being currently employed as a CHW, a resident of Appalachia for at least 12 months, and primarily working with adults. Surveys took between five to 30 minutes to complete. Organization directors sent a recruitment email with study details and survey link. CHWs who completed the surveys were invited to participate in an in-depth interview. Interviews were conducted over the phone, recorded, and transcribed for analysis. The interviews lasted 41–54 minutes. Survey participants received a $50 gift card incentive, and interview participants received an additional $75 gift card.

### Measures

Two instruments were used for this study: an online survey, using Qualtrics (https://www.qualtrics.com) and an in-depth interview semi-structured question guide (Appendix 1). Survey instruments were selected to explore associations between cultural competency, knowledge about nutrition-related community programs, and experiences influencing professional characteristics. The 64-item survey was used to collect information on cultural competency, childhood socioeconomic status (to examine congruency with community being served),^7^ professional experiences (volunteer, work, or training to examine how CHWs apply knowledge from other professional or volunteer activities), knowledge and referrals to food assistance programs, perceptions of their ability to obtain food security resources for their clients, and demographics. The Cultural Competence Assessment (CCA) tool measures Cultural Awareness and Sensitivity (CAS) and Culturally Competent Behaviors (CCB).^8^ Attitudes and beliefs items were assessed on a 7-point Likert scale ranging from “strongly agree” to “strongly disagree” and frequency of from “always” to “never.” Semi-structured in-depth interviews had 31 questions and were developed using the Rural Health Nutrition Care Model and Tool for Health and Resilience in Vulnerable Environments.^9^,^10^ Questions were designed to explore the four nutrition and food security factors relative to access and resources, sociocultural characteristics, traditional foods, and health behaviors defined by the Rural Health Nutrition Care Model.^9^

### Data Analysis

#### Survey

Statistical analysis of the surveys included descriptive statistics and reliability testing of validated questionnaires using Cronbach’s alpha.

#### Interview

In-depth interviews were coded by the primary investigator and a trained research assistant using thematic analysis.^11^ This method includes steps such as familiarizing oneself with the data, generating initial codes, searching for themes, reviewing themes, defining and naming themes, and producing the report. Coders independently coded and met subsequently to establish a consensus of themes. Final coding was facilitated through NVivo 12 software^12^ to refine the themes and subthemes.

## RESULTS

### Results from Survey

Fourteen CHWs completed the survey, averaging an age of 52.9 (+15.9 SD); all identified as female, and most identified as non-Hispanic white (n=13). The highest levels of education completed were high school (n=5), some college (n=3), and college (n=6). Only one had worked as a CHW less than one year; the remaining had between one to three years (n=5), four to seven years (n=4), and eight years or more (n=4). The Cronbach’s alpha for the cultural competence assessment scale and subscales ranged between 0.82 to 0.89, indicating acceptable reliability. The overall cultural competence assessment scale score was an average of 5.21 (+0.75 SD); the cultural awareness subscale score was an average of 5.10 (±0.43 SD); the cultural competency behavior subscale score was an average of 5.32 (±1.21 SD). CHWs reported the frequency and ability to provide guidance for obtaining food bank boxes (n=14), church pantry food boxes (n=14), and summer feeding programs for children (n=13) (Table 1). A program director with 27 CHWs reported over 7,000 community touchpoints in 2018. The second director had 21 CHWs but did not report community touchpoints.

#### Results from Interviews

CHWs identified current or prior paid or volunteer roles that helped them as CHWs. These roles included paid positions in medical fields like hospice, clinical secretary, dentist’s office, school systems, health departments, or teaching diabetes, cooking, or nutrition courses. All CHWs reported volunteering and most (n=4) reported volunteering with multiple programs such as food banks (n=2), church programs (n=3), and programs for youth and children (n=4).

#### Theme 1: Unique Barriers to Addressing Hunger

Non-custodial grandparents raising grandchildren was reported as a factor that made addressing hunger in rural Appalachian communities more complex (n=5). CHWs reported that 20–50% of grandchildren in their communities live with their grandparents and noted the extra strain placed on grandparents. Generational differences were reported regarding food choices. “Older generations” were more likely to grow a garden, cook their meals, and consume more vegetables. In comparison, “younger generations” were more likely to consume convenience foods, fast foods, and foods requiring minimal cooking skills. CHWs reported barriers to gardening such as lack of skill in the younger generations, lack of ability due to age or disability in the older generations, and lack of land.

#### Theme 2: Unique Solutions to Addressing Hunger

CHWs identified unique solutions to addressing hunger in their communities like community agency and gardening (n=6 for both). Community agency involved individuals helping to reduce food insecurity through actions like lending food or money, cooking meals for neighbors or family members, carpooling, and picking up groceries or emergency food resources for others. Only two CHWs reported knowing of programs in the community that supported gardening.

## DISCUSSION

This study begins to explain what contributes to CHWs’ moderately high cultural competence scores (CCA score of 5.21)^13^ and their perceptions about their ability to guide community members in obtaining nutrition and food security resources. Our findings are consistent with other rural communities regarding reliance on food assistance programs and resources.^14^ Due do deep community ties, the current study contributes the insights from CHWs on how they connect their community to food assistance programs and resources and can provide community-specific solutions to address hunger.^15^

### Unique Barriers and Solutions to Addressing Hunger

One unique barrier is about non-custodial grandparents. CHWs explained the familial reliance on caregiving and food. Non-custodial grandparents have limited means and lack legal guardianship required to access programs like SNAP for grandchildren.^16^ They reported that parents may resist relinquishing guardianship due to emotional factors like guilt or a desire to retain benefits for themselves. Therefore, CHWs identified gardening as a potential solution. CHWs reported that older adults had more knowledge about gardening but were physically unable to garden. Further, they are not passing down this knowledge due to the increased availability of convenience foods which younger generations tend to choose. Research suggests that a decline in traditional skills such as gardening and food preservation has increased the reliance on food assistance programs.^17^ More research about intergenerational activities related to food, nutrition, and resource-sharing may be considered in development of food and nutrition security programs for low-income, multigenerational households.

The deep knowledge that CHW’s have provide may be useful for developing targeted training and programming foster community agency, such as strengthening gardening skills.^18^

CHWs identified community agency as a key solution to hunger in rural Appalachia, emphasizing food sharing, carpooling for groceries, and expanding home and community gardens to improve access to fresh produce.^17^

The noted study limitations are as follows: (1) a small sample from only two of 13 Appalachian states which limits application to the region; (2) self-selection bias; (3) survey instruments were not previously validated with CHWs; and (4) data were collected before COVID-19, which increased interest in home gardening.^19^ Due to the small sample size, no analyses were completed to explore associations between cultural competency, socioeconomic status, knowledge about nutrition-related community programs, and experiences that influence professional characteristics. However, barriers identified were consistent with the literature, such as lack of access to immediate food resources, transportation, misuse of food assistance, inconsistent availability and affordability of food, addiction, disabilities, housing insecurity, multiple families in one household, and health problems.^20^

## CONCLUSION

Future research should focus on including CHWs as partners in developing policies and programs; additionally, education initiatives related to food and nutrition security are key to engaging communities and implementing practical solutions. Policy changes should also be considered to support community-driven solutions such as carpooling initiatives and the expansion of home and community gardens. CHWs have a moderate degree of cultural competence and extensive knowledge of unique barriers, solutions, and food assistance programs in their communities.

**SUMMARY BOX**

**What is already known about this topic?**

Community health workers (CHWs) are trusted members of their communities and possess cultural competence that helps address social determinants of health, such as food insecurity.

**What is added by this report?**

This study provides specific insights into CHWs’ professional characteristics and their culturally sensitive practices for addressing food insecurity in rural Appalachia, highlighting barriers like generational challenges and solutions that foster community agency.

**What are the implications for future research?**

Future research should engage with and leverage CHWs’ cultural competence to design community-based interventions that improve food and nutrition security in rural communities.

## Supplementary Information



## Figures and Tables

**Table 1:** Community Health Workers’ Self-Reported Ability to Provide Guidance on Food Assistance Programs

I am able to provide guidance to families to obtain access to:	Always n (%)	Somewhat/ Very Often n (%)	Often n (%)	Some/ Few Times n (%)	Never n (%)	Not Sure n (%)
Supplemental Nutrition Assistance Program (SNAP)	6 (42.9)	6 (42.9)		2 (14.3)		
Farmers Market Vouchers	6 (42.9)	5 (35.7)		2 (14.3)	1 (7.1)	
Mobile Farmers Markets	4 (28.6)	5 (35.7)		2 (14.3)	1 (7.1)	2 (14.3)
Food Bank Boxes	7 (50.0)	5 (35.7)	2 (14.3)			
Church Pantry Food Boxes	7 (50.0)	5 (35.7)	2 (14.3)			
Summer Feeding Programs for Children	8 (57.1)	3 (21.4)	2 (14.3)		1 (7.1)	
Garden Seed Programs	4 (28.6)	4 (28.6)		3 (21.4)	1 (7.1)	2 (14.3)
Community Gardens	4 (28.6)	2 (14.3)		3 (21.4)	2 (14.3)	3 (21.4)
