# Supplementary material for: Leveraging Community Health Worker’s Deep Community Knowledge to Address Food and Nutrition Security in Appalachia
Source: J Appalach Health. 2025 Dec 1;7(4):89–99. doi: 10.13023/jah.0704.05 (PMC13239644; doi:10.13023/jah.0704.05)
Supplement: Supplementary file 1 [file 7.4.5_Scottetal_Additionalfile.docx]

**Qualitative Interview Question Guide based on the four factors of the Rural Health Nutrition Care Model**

**Factor 1. Access and Resources (finances, location/ distance/food insecurity)**

1. Among the families you help, what does hunger look like? Prompt: You can use an example of a family to describe how hunger affects the family as a whole or each individual member.
2. What are some examples of challenges you face when trying to address hunger with families? Prompt: Are there some examples from families who you were not able to help and why were you not able to help them?
3. When a family is in need of food, what are some ways that their family, friends or neighbors help?
4. When a family is in need of food, what are resources to obtain foods?
5. Are healthy products available and affordable in our community?
6. Can we affordably and efficiently get to where we need to be?
7. Can I safely drink the water and breathe the air in my neighborhood?
8. Tell us about your experience in providing services related to food and nutrition for clients.
9. What if any barriers can you identify regarding providing food and nutrition for your clients?
10. What issues impact the delivery of food and nutrition to your clients?
11. Describe lessons you have learned about delivering food and nutrition to your clients.
12. What influences the food choices of your clients?

**Factor 2. Sociocultural Characteristics (Resistance to change, religion, family, storytelling)**

1. Do people know and trust each other?
2. What other positions, volunteer or paid, have you held that have helped you as a CHW [or program assistant]? Prompt: these positions can be through your church, children’s schools,
3. Can you give me an example of something that you learned in the role as [participant stated] that helps you in your current role?
4. What do you think is unique about providing nutrition and food services to people living in Appalachia?
5. What are some major nutrition and food service concerns that you see among your clients?

**Factor 3. Traditional Foods (Familiar foods, dietary patterns and pathways, family meals)**

1. What does hunger look like in your community?
2. Where do people obtain most of their food? (Prompt: in town, out of town, specific shop in neighborhoods, etc.)
3. Have you noticed if people in your community have gardens where they live? What types of foods/plants do people garden?
4. What does a typical meal for a family look like? Who prepares the meal, who eats that meal and where is it eaten?
5. What is an example of a solution to hunger or food insecurity that you have seen your clients use to solve their own or other’s?
6. What is an example of a solution to any issue that you have seen your clients use to solve their own or other’s issue?
7. What is an example of how your clients have overcome a major barrier? (Related to food, education, employment, healthcare, etc.)

**Factor 4. Health Behaviors (Preventative care, substance abuse, self-management, chronic disease)**

1. Who does most of the cooking for your clients? If themselves, Prompt: Who do your clients cook for?
2. What skills, knowledge or techniques do you believe you clients would benefit from learning or practicing to reduce food insecurity and hunger?
3. What other health problems make food insecurity and hunger worse?
4. What health problems does food insecurity and hunger make worse?
5. What is an example of a change that a client or family has made to improve their access to foods?
6. What individuals are most knowledgeable about food insecurity and hunger in the community?
7. What individuals do your clients trust the most to talk to about food insecurity and hunger?
